# Supplementary material for: Detecting topology freezing transition temperature of vitrimers by AIE luminogens
Source: Nat Commun. 2019 Jul 18;10:3165. doi: 10.1038/s41467-019-11144-6 (PMC6639363; doi:10.1038/s41467-019-11144-6)
Supplement: Supplementary file 1 — Supplementary Information [file 41467_2019_11144_MOESM1_ESM.pdf]

Supplementary Information

Detecting topology freezing transition temperature of vitrimers by AIE luminogens

Yang Yang[1], Shuai Zhang[1], Xiqi Zhang[2], Longcheng Gao[3]*, Yen Wei[1,4], Yan Ji[1]*

Correspondence to: jiyan@mail.tsinghua.edu.cn; lcgao@buaa.edu.cn

[1]The Key Laboratory of Bioorganic Phosphorus Chemistry & Chemical Biology (Ministry of Education), Department of Chemistry, Tsinghua University, Beijing 100084, China

[2]CAS Key Laboratory of Bio-inspired Materials and Interfacial Science, Technical Institute of Physics and Chemistry, Chinese Academy of Sciences, Beijing 100190, China

[3]Key Laboratory of Bio-Inspired Smart Interfacial Science and Technology of Ministry of Education, School of Chemistry, Beihang University, Beijing 100191, China

[4]Department of Chemistry, Center for Nanotechnology and Institute of Biomedical Technology, Chung-Yuan Christian University, Chung-Li 32023, Taiwan, China

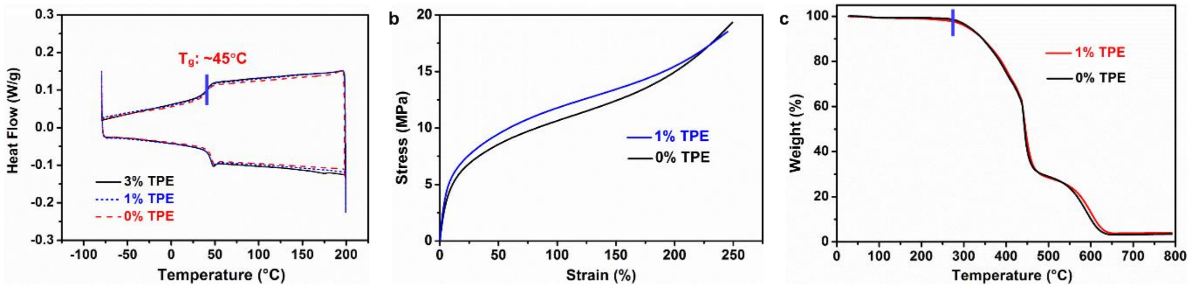

Supplementary Figure 1. (a) DSC traces of both heating/cooling (rate of 5 ℃/min) of the epoxy vitrimers doped with 3 wt%, 1 wt% and 0 wt% TPE. T_g of three samples is ~45 ℃. And adding TPE does not affect T_g obviously. (b) Stress-strain curves of epoxy vitrimers doped with 1 wt% and 0 wt% TPE measured by DMA. The mechanical strengths of both samples are similar. (c) TGA curves of epoxy vitrimers doped with 1 wt% and 0 wt% TPE under air atmosphere (Heating rate: 20 ℃/min). Onset of decomposition under air atmosphere is about 275 ℃. The thermal stabilities of both samples are similar.

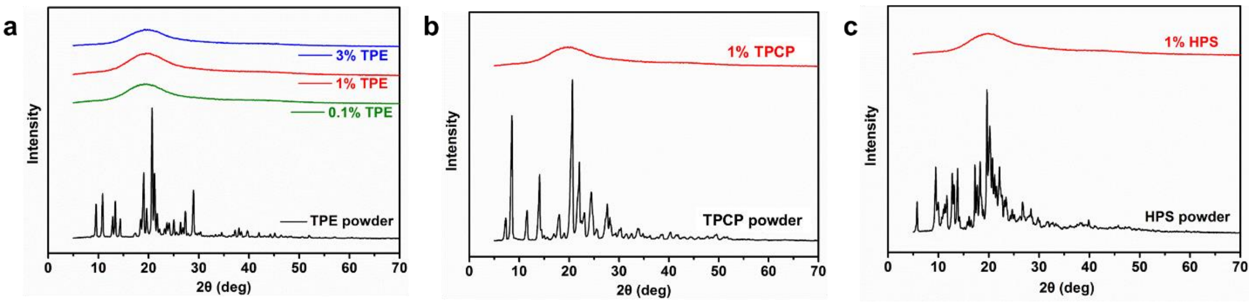

Supplementary Figure 2. (a) XRD curves of TPE-doped (containing 3 wt%, 1 wt% and 0.1 wt% TPE) epoxy vitrimers and TPE powder. (b) XRD curves of 1 wt% TPCP-doped epoxy vitrimer and TPCP powder. (c) XRD curves of 1 wt% HPS-doped epoxy vitrimer and HPS powder.

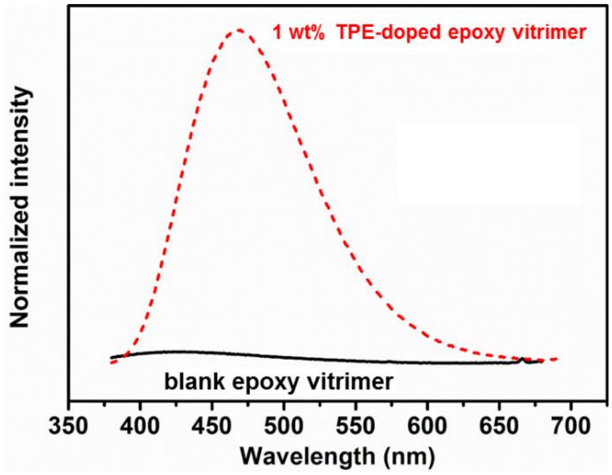

Supplementary Figure 3. Fluorescence spectra of 1 wt% TPE-doped epoxy vitrimer and blank epoxy vitrimer at room temperature (Excitation: 365 nm).

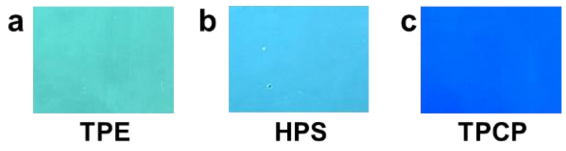

Supplementary Figure 4. Fluorescent images of 1 wt% TPE-doped (a), 0.1 wt% HPS-doped (b) and 1 wt% TPCP-doped (c) epoxy vitrimers under UV radiation (365 nm).

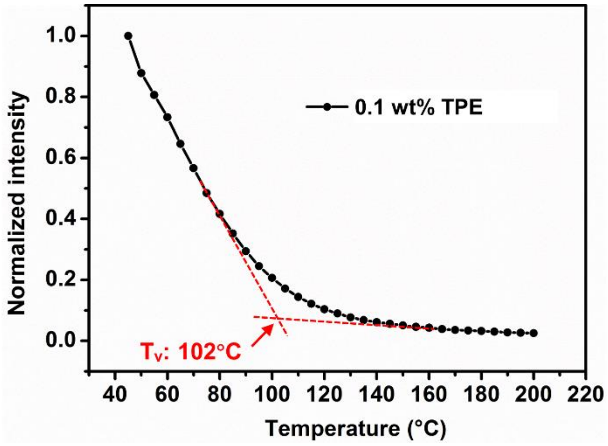

Supplementary Figure 5. The fluorescence intensity plot of 0.1 wt% TPE-doped epoxy vitrimer at a series of temperature at 470 nm (Excitation: 365 nm). All the fluorescence intensities are normalized to 45 ℃.

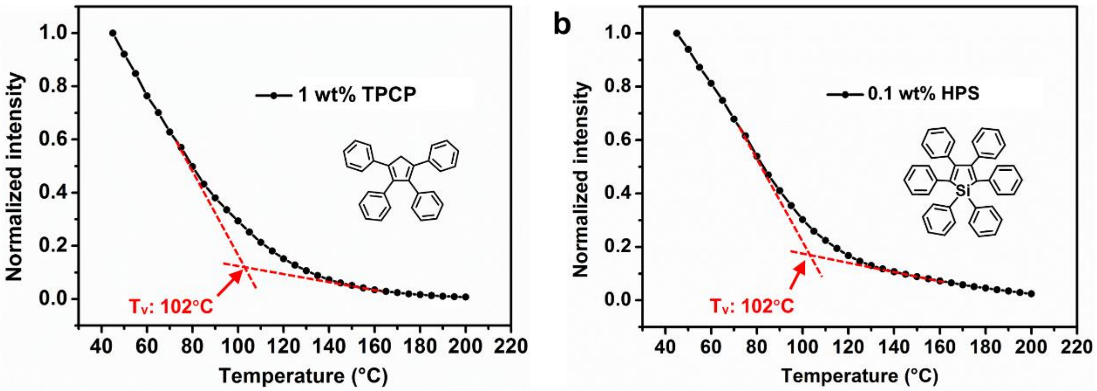

Supplementary Figure 6. (a) The fluorescence intensity plot of 1 wt% TPCP-doped epoxy vitrimer at a series of temperature at 440 nm (Excitation: 330 nm). All the fluorescence intensities are normalized to 45 ℃. (b) The fluorescence intensity plot of 0.1 wt% HPS-doped epoxy vitrimer at a series of temperature at 490 nm (Excitation: 407 nm). All the fluorescence intensities are normalized to 45 ℃.

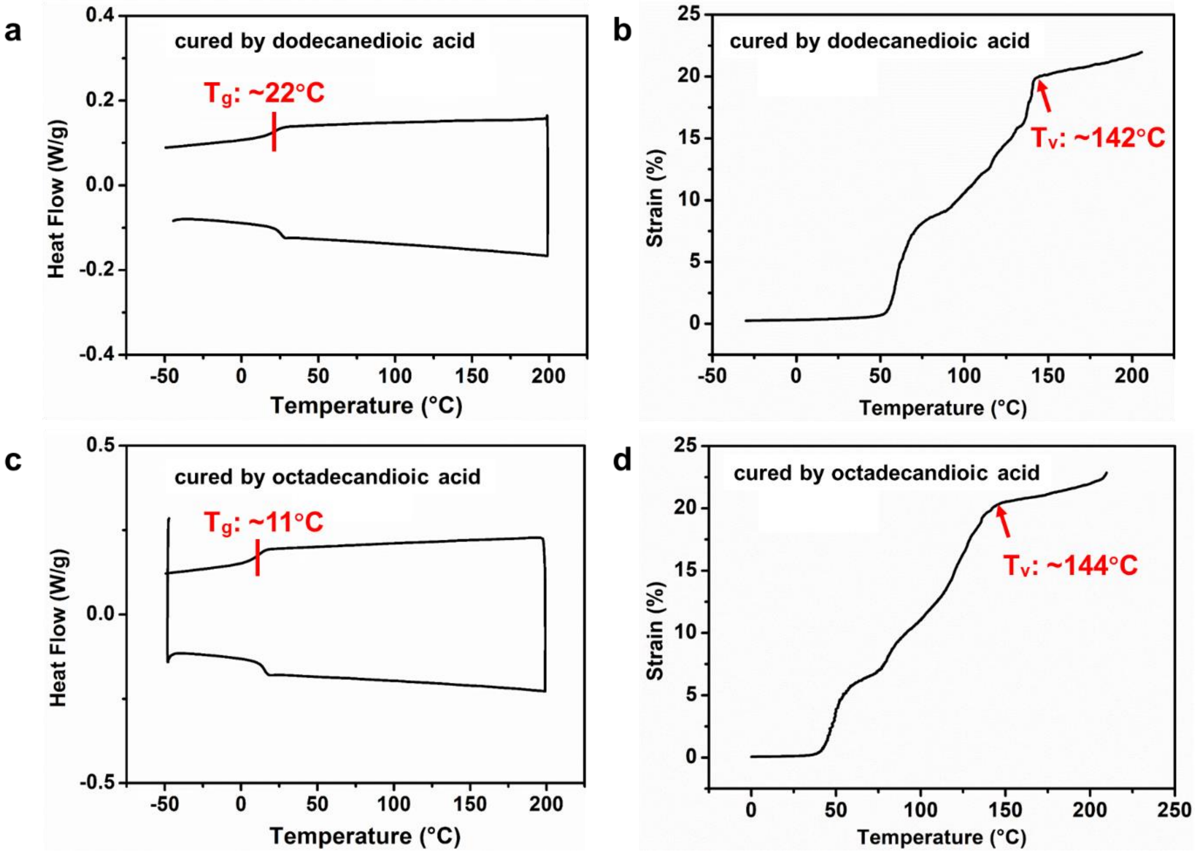

Supplementary Figure 7. Thermal characterizations of blank epoxy vitrimers cured by octadecanedioic acid and dodecanedioic acid. (a) DSC trace of both heating/cooling (rate of 5 ℃/min) of blank epoxy vitrimer cured by dodecanedioic acid. (b) Dilatometry test of blank epoxy vitrimer cured by dodecanedioic acid at a rate of 5 ℃/min. (c) DSC trace of both heating/cooling (rate of 5 ℃/min) of blank epoxy vitrimer cured by octadecanedioic acid. (d) Dilatometry test of blank epoxy vitrimer cured by octadecanedioic acid at a rate of 5 ℃/min.

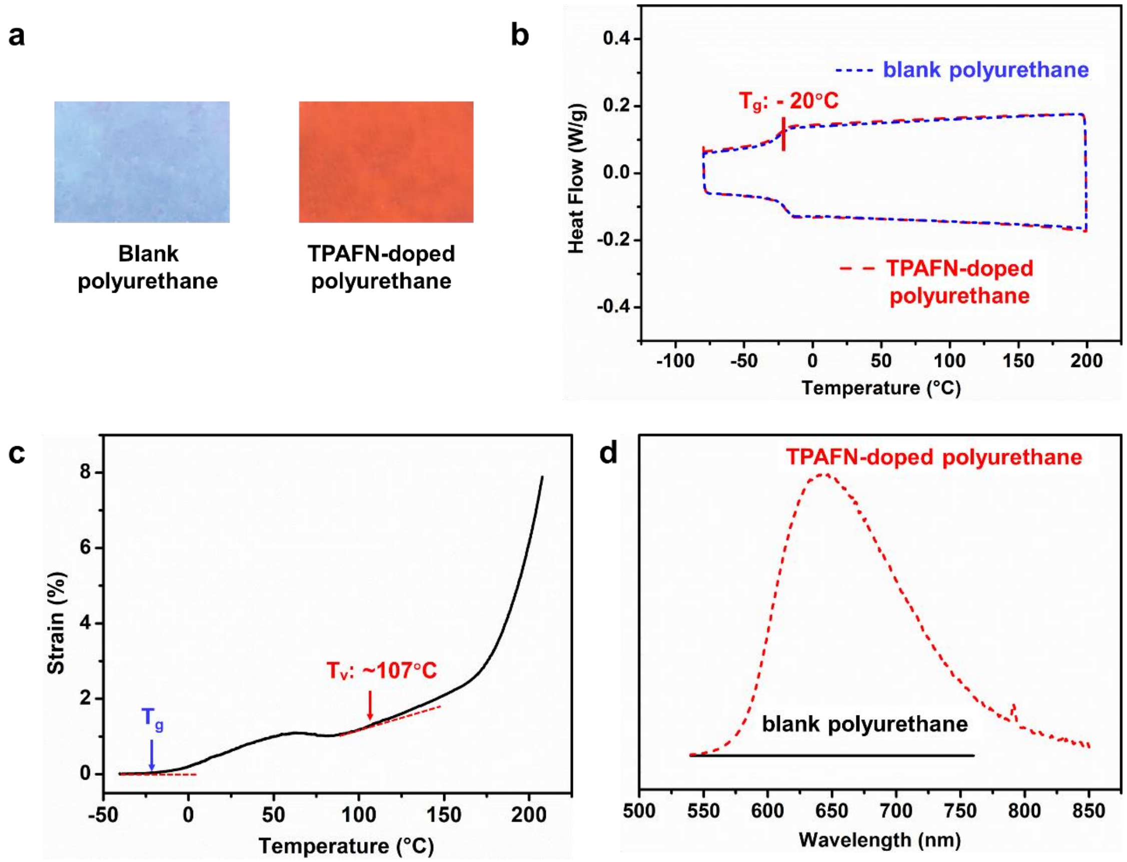

Supplementary Figure 8. Thermal and fluorescent characterizations of TPAFN-doped polyurethanes. (a) Fluorescent images of 0.1 wt% TPAFN-doped polyurethane and blank polyurethane under UV radiation (365 nm). (b) DSC traces of both heating/cooling (rate of 5 ℃/min) of 0.1 wt% TPAFN-doped polyurethane and blank polyurethane. T_g of both samples is ~ -20 ℃. (c) Dilatometry test of blank polyurethane heated from 25 to 200 ℃ at a rate of 5 ℃/min. It shows T_v is about 107 ℃. (d) Fluorescence spectra of 0.1 wt% TPAFN-doped polyurethane and blank polyurethane at room temperature (Excitation: 527 nm).

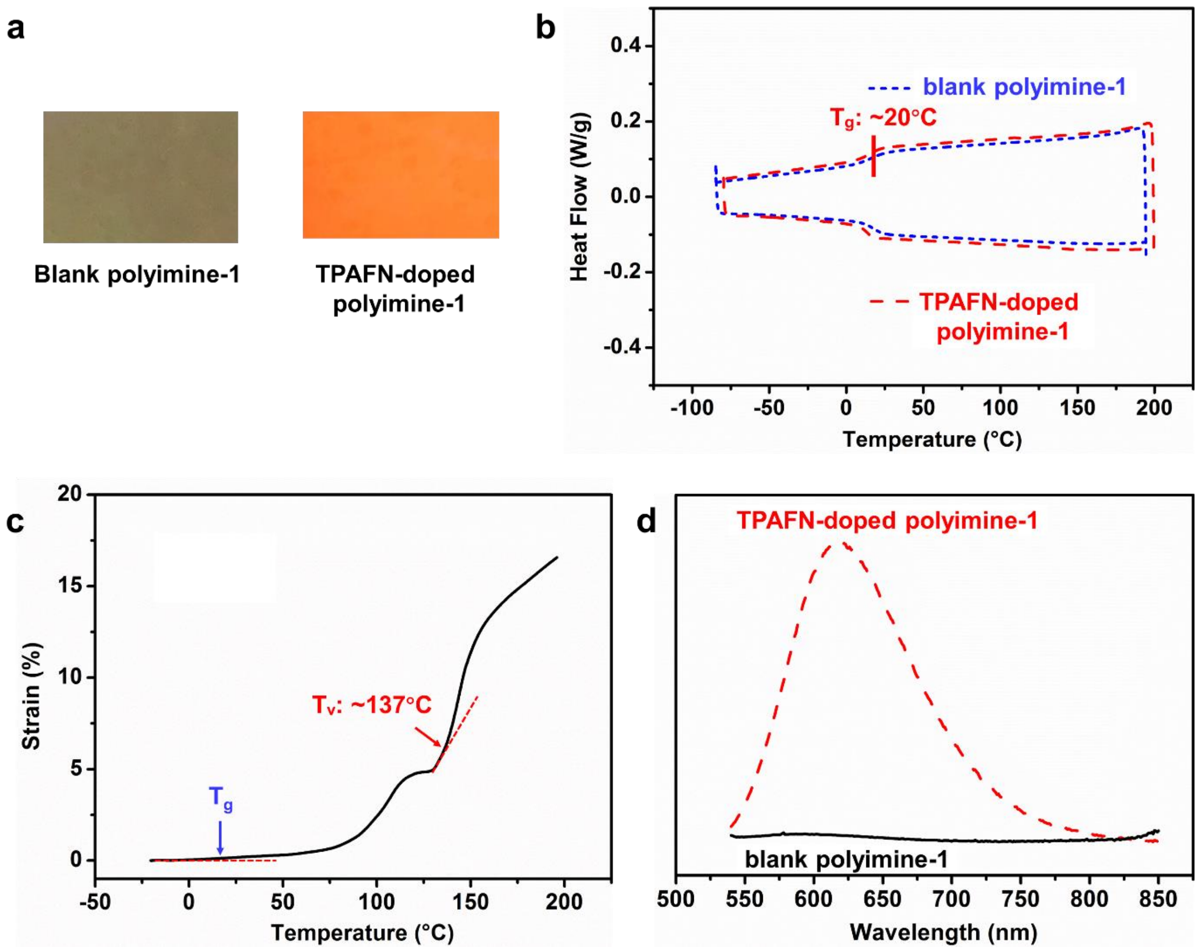

Supplementary Figure 9. Thermal and fluorescent characterizations of TPAFN-doped polyimime-1. (a) Fluorescent images of 0.5 wt% TPAFN-doped polyimine-1 and blank polyimine-1 under UV radiation (365 nm). (b) DSC traces of both heating/cooling (rate of 5 ℃/min) of 0.5 wt% TPAFN-doped polyimine-1 and blank polyimine-1. T_g of both samples is ~ 20 ℃. (c) Dilatometry test of blank polyimine-1 heated from 25 to 200 ℃ at a rate of 5 ℃/min. It shows T_v is about 137 ℃. (d) Fluorescence spectra of 0.5 wt% TPAFN-doped polyimine-1 and blank polyimine-1 at room temperature (Excitation: 527 nm).

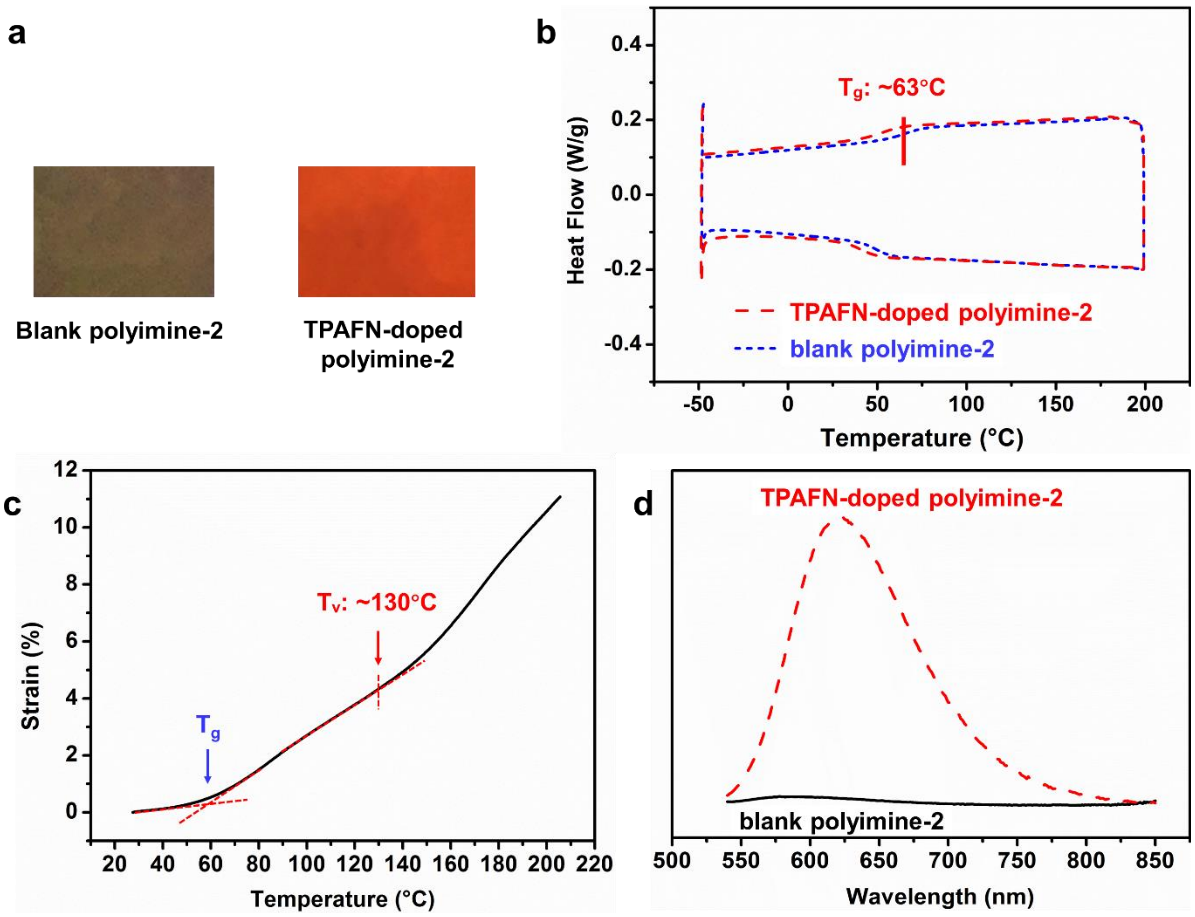

Supplementary Figure 10. Thermal and fluorescent characterizations of TPAFN-doped polyimime-2. (a) Fluorescent images of 0.5 wt% TPAFN-doped polyimine-2 and blank polyimine-2 under UV radiation (365 nm). (b) DSC traces of both heating/cooling (rate of 5 ℃/min) of 0.5 wt% TPAFN-doped polyimine-2 and blank polyimine-2. T_g of both samples is ~ 63 ℃. (c) Dilatometry test of blank polyimine-2 heated from 25 to 200 ℃ at a rate of 5 ℃/min. It shows T_v is about 130 ℃. (d) Fluorescence spectra of 0.5 wt% TPAFN-doped polyimine-2 and blank polyimine-2 at room temperature (Excitation: 527 nm).
